# Inhaled corticosteroid influence toll like receptor 2 expression in induced sputum from patients with COPD

**DOI:** 10.1186/2213-0802-1-7

**Published:** 2013-03-19

**Authors:** Haixing Zhu, Yuheng Shi, Wei Tang, Guocao Shi, Huanyin Wan

**Affiliations:** 1 Department of Respirology, Ruijin Hospital affiliated to Jiaotong University School of Medicine, Shanghai, 200025 China; 2 Department of Respirology, No.3 people’s hospital affiliated to Jiaotong University School of medicine, Shanghai, 200133 China

**Keywords:** COPD, TLR2, Induced sputum, Macrophage

## Abstract

**Background:**

The link between long-term ICS therapy and respiratory infection in COPD patients is controversial. We investigated the effect of long-term use of inhaled corticosteroid on Toll like receptor 2 (TLR2) expression in induced sputum from COPD patients.

**Methods:**

51 patients were divided into two groups according to their treatment history: long-term ICS treatment group (patients who have used ICS (equivalent to Fluticasone Propionate (FP) ≥ 500 ug/day for more than 1 year) (n = 21) and ICS naive group (who have never routinely used ICS before, n = 29). In their induced sputum, we tested TLR2 extracellular and intracellular expression on macrophages using flowcytometry. TLR2 and tumor necrosis factor αmRNA expression were also evaluated by real-time PCR.

**Results:**

TLR2 extracellular expression on the macrophages from induced sputum in long-term ICS treatment group was lower than the ICS naïve group (13.69% ± 1.17% vs 20.12% ± 4.37%, p = 0.019). TLR2 intracellular expression in the macrophages, the TLR2 and TNFαmRNA in the induced sputum also showed a trend towards decreased endpoint in ICS long-term treatment group compare to ICS naïve group but did not reach significance. TLR2 extracellular and TLR2 intracellular expression were strongly related (r = 0.645, p = P = 0.017) as well as TNFαmRNA and TLR2 mRNA expression (r = 0.894, p = 0.0001).

**Conclusion:**

Long-term use of ICS may have negative influence on TLR2 expression in the airway of severe COPD patient.

**Electronic supplementary material:**

The online version of this article (doi:10.1186/2213-0802-1-7) contains supplementary material, which is available to authorized users.

## Background

Inhaled corticosteroids (ICS) are recommended by current guidelines for use in the treatment of chronic obstructive pulmonary disease (COPD) for those with severe disease, defined as a forced expiratory volume in first second (FEV1) <50% predicted and repeated exacerbations. Recent large prospective trials have reported an increased incidence of pneumonia in COPD patients using ICS on daily basis. This has raised concern about long term safety of ICS therapy. The link between ICS therapy and pneumonia remains controversial [1].

COPD patients are susceptible to various pathogens. Toll like receptor 2 (TLR2) is a critical component in the host innate immune response to infections. TLR2 signaling has been proposed to be involved in the pathogen recognition process during the infection of Streptococuus, mycoplasma, chlamydia, virus, which are the common pathogens that cause exacerbations and pneumonia in COPD [2–5]. Because of this, we hypothesized that long-term use of inhaled corticosteroid may down-regulate TLR2 expression therefore reducing microbial recognition. In this study, we investigated severe COPD patients who receiving long term ICS treatment and compared the expression of TLR2 in the macrophages from induced sputum.

## Methods

### Subjects

The ethnic review board of Ruijin hospital affiliated to Shanghai Jiaotong University school of medicine approved the study, and informed consent was obtained from all subjects. According to the Global Initiative for Chronic Obstructive Lung Disease (GOLD) and the Chinese guideline on Chronic Obstructive lung disease, 51 severe stable COPD patients who were in stage III and IV with FEV1 less than 50% predicted value were investigated in this study. Patients who had an exacerbation in the previous 6 weeks or who were hospitalized in the previous 3 months were excluded from the study. Patients on oral steroids or who concomitantly suffered from bronchiactasis, chronic fungus infections and asthma were also excluded.

### Induced sputum processing

Spirometry was recorded 15 min after inhalation of 200ug salbutamol via a metered-dose inhaler. Subjects inhaled 3% saline at room temperature, nebulized via an ultrasonic nebulizer (ShuangYu® Ultrasonic nebulizer, China) at maximum output for 20 minutes. Subjects were encouraged to cough deeply. Sputum was coughed into polypropylene pots. Saliva was discarded. If bothersome symptoms occur, the nebulization should be stopped and the subject treated with salbutamol. After the sputum induction, spirometry was repeated. If the FEV1 falls by more than 20%, the subject was required to wait until it had returned to baseline value.

### Sputum processing

The sputum samples were kept at 4°C for not more than 2 h prior to further processing. The portion of the sample for cell counting was diluted with 8 ml 1% dithiothreitol (DTT) (shanghai ZiYi reagent, China) and gently vortexed. The resultant cell suspension was then filtered through 400 μm sieves. After centrifuge with 1500 rpm for 10 minutes, cell pellet were washed twice with PBS containing 2% FCS, then resuspended in PBS. Total cell counting was carried out on a hemacytometer and cell viability was calculated by using the trypan blue. Cells were adjusted to a concentration of 1 × 10^5^/ml and use 0.5 ml cell suspension for flow cytometry staining. The remaining cells were centrifuged and resuspended to be used in later RT-PCR testing.

### Flowcytometry analysis

For extracellular staining, 10 ul mouse anti-human CD14 FITC monoclonal antibody and 10 ul of mouse anti-human TLR2 PE monoclonal antibody or relative IgG antibody were added, incubated for 1 hour. For intracellular staining, 10 ul mouse anti-human CD14 FITC monoclonal antibody was added to the cell suspension and incubated for 1 hour in room temperature first. The sample was then fixed with 4% paraformaldehyde for 20 minutes. The cells were resuspended with 200 ul 0.1% saponin for 10 minutes. Mouse anti-human TLR2 PE monoclonal antibody 10 ul or relative IgG antibody were added to the sample and incubated for 1 hour. (Antibodies all from eBioscience, USA) Cells were then resuspend in PBS and acquired on a flow-cytometry. The expression of extra and intracellular TLR2 from macrophages was identified by both positive staining for TLR2 and CD14 staining. (BECTON-DICKINSCN FACS Calibur, BD Biosciences) Cellquest software was used for the analysis (eBioscience, USA).

### RT-PCR

We examined the expression of TLR2mRNA and TNFαmRNA expression in the induced sputum from severe COPD subjects of long term ICS therapy compared to subjects on no long term therapy. Among all the sputum samples, 12 in ICS naïve group and 10 in ICS group have the remaining cells for RT-PCR testing after flowcytometry testing. Target gene expression was analysed using quantitative real-time PCR. Briefly, RNA was extracted and reverse-transcribed to cDNA. The oligonucletoide primers for TLR2 were: upper: 5^′^ GGG TCT TGG GGG TCA TCA T 3^′^; lower: 5^′^ CAG AGC CTG GAG GTT CAC A 3^′^; Primes for TNFαwas: upper:5^′^ AGA GGG AGA GAA GCA ACT ACA G 3^′^; lower: 5^′^ CCG TGG GTC AGT ATG TGA G 3^′^. Reactions were characterized by comparing the threshold cycle (CT) values. Taqman qPCR probes for TLR2 and TNFαmRNA were purchased in kit form, combined with the reference gene eukaryotic 18S ribosomal RNA in dupliate real-time PCRs chains (ABI prism7900 Real Time PCR Machine, Applied Biosystems, USA) and results were calculated using 2-ΔCt relative to the housekeeping gene (18S) and an internal calibrator. (2-ΔCt = the difference in threshold cycles for the test gene and beta actin).

### Statistical analysis

Expression levels are expressed as means +/− SD. Comparisons between the two groups were performed by SPSS 11.0 version software of independent-samples T test. Use Pearson correlation test for determine the association between mRNA level and TLR2 expression. A p value < 0.05 was considered significant.

## Results

### General characteristics of subjects

All of our subjects were male and smokers or ex-smokers. We divided these patients into two groups according to the treatment history: Long term ICS treatment group (with fluticasone propionate (FP) use ≥ 500 ug/day for more than 1 year) and ICS naïve group (without routine ICS use). In ICS treatment group, the mean ICS treatment duration was 1.65 ± 0.75y (1–3.5 years). Most patients received FP 250 ug twice daily inhaled. (20 in 21 subjects, the other subject received 500 ug FP twice daily inhaled treatment). The demographic data in both groups are shown in Table 1. Except the ICS use duration, the demographic characteristics data between these two groups showed no statistical difference.

**Table 1:** **Demographic characteristics of study participants**

	ICS group (n = 21)	ICS naïve group (n = 29)	P value
Age (years)	66 (79–54)	68 (84–56)	0.86
Gender (female/male)	0/21	0/29	1
Duration of COPD (year)	5 (1–25)	4 (0.5–21)	0.15
Smokers/ex-smokers	9/21	14/29	-
Smoking (pack/year)	56 (82–20)	55 (60–20)	0.76
BMI (kg/m^2^)	27 (35–20)	26 (38–19)	0.23
Post BD FEV1 (%predicted)	43% (15–50)	48% (17–49)	0.47
FEV1/FVC	32% (12-46%)	36% (20-54%)	0.13
MRC† Dyspnoea Score	2 (2–3)	2 (2–3)	0.61
Hospitalization in the previous year (number)	0.5 (0–1)	0.4 (0–1)	0.14
Pneumonia in the previous 3 years (number)	0.6 (0–1)	0.5 (0–1)	0.07
Non of ER visit because of exacerbation of COPD in the previous year (number)	1.1 (1–3)	1.0 (1–2)	0.09

†: MRC = Modified Medical Council.

Data represents: mean (range).

### Impairment of TLR2 expression on macrophage in COPD patient who treated with ICS

**Figure 1:**
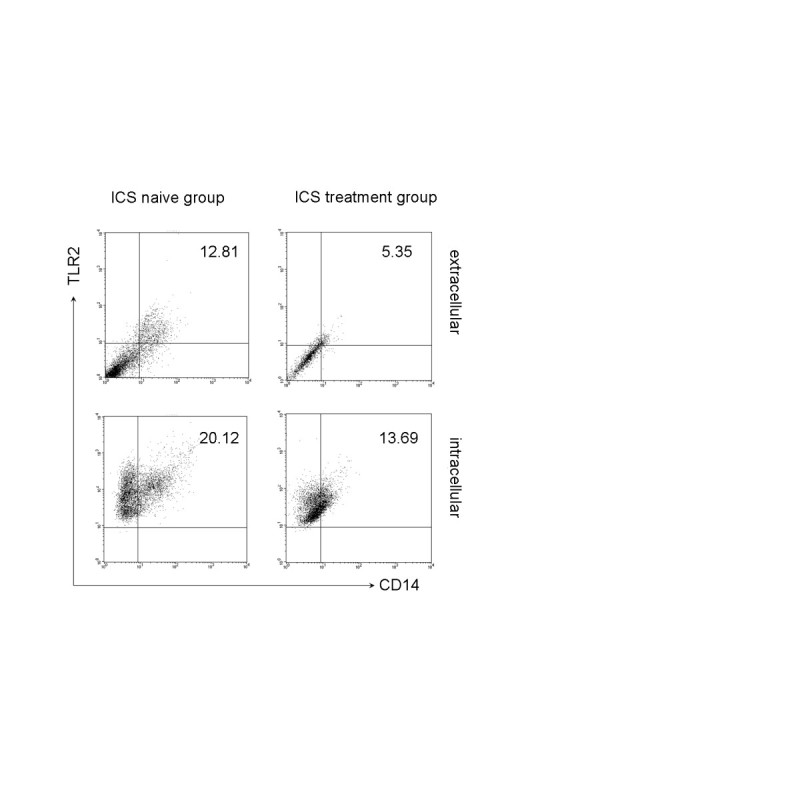
**Intracellular and surface expression of TLR2 on macrophages in induced sputum.** Flowcytometry results from typical subjects in the two groups. Right upper quardrant presents the events of the percentage of both positive in TLR2 and CD14 staining. From upper to bottom: extracelluar expression of TLR2, intracellular expression.

**Figure 2:**
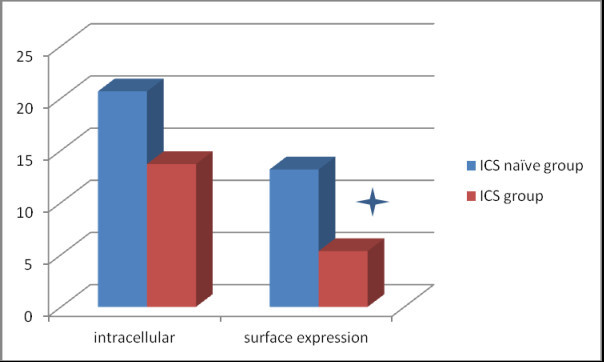
**Comparison of TLR2 surface (extracellular) and intracellular expression in the induced sputum macrophages between ICS naïve group and ICS long term treatment groups.** Bar presents the mean value. *p < 0.05 comparison between the two groups.

The expression of TLR2 was identified as both extracellular (surface expression) and intracellular in the induced sputum macrophages (CD14+ TLR2+ cell) (Figure 1). In the ICS naïve group, the TLR2 intracellular expression was 20.12% ± 4.37% and extracellular expression was 12.81% ± 4.89%. In the ICS treatment group (patients received ICS treatment more than one year and ≥500 ug/day fluticasone propionate), the TLR2 intracellular expression was 13.69% ± 1.17% and extracellular expression was 5.35% ± 1.67%. The extracellular expression of TLR2 on induced sputum macrophages in ICS treatment group was significantly lower than ICS naïve group (p = 0.019) (Figure 2).

### TLR2 and TNFαmRNA expression in induced sputum from COPD subjects

Constitutive mRNA expression was measured for TLR2 and TNFα (tumor necrosis factor α) from induced sputum in severe COPD patients. Values have been normalized to the expression of the house keeping gene. The TLR2 mRNA and TNFαmRNA expression slightly decreased in ICS treatment group compare to ICS naïve group but this did not reached statistical significance. (Table 2) Pearson correlation test was used to determine the association between mRNA level and TLR2 expression as well as TLR2 mRNA and TNFαmRNA expression. A statistically significant correlation between intracellular and extracellular expression of TLR2 in both groups was observed (r = 0.645, p = 0.017). A stronger correlation between TLR2 mRNA and TNFαmRNA expression was also observed (r = 0.894, p = 0.000).

**Table 2:** **TLR2 mRNA and TNFαmRNA expression in induced sputum from COPD patients**

mRNA expression	ICS naïve group	ICS group	p value
TNFα	8.18 ± 1.49	6.92 ± 2.23	0.31
TLR2	5.13 ± 1.41	3.64 ± 1.61	0.37

Data represents: Mean ± SD.

## Discussion

Recurrent airway infections are the leading cause of COPD exacerbation, which are also strongly related to the prognosis of COPD. COPD patients are susceptible to bacterial and virus infections probably due to local airway immunity impairment [6]. Innate immunity is the first line of defense against infection and toll-like receptor mediated recognition of pathogens is essential. Impairment of the TLRs leads to an increase in pathogen susceptibility in COPD patients. For example, TLR2 is involved in the host immunity during pulmonary infection with pathogen like Chlamydia pneumoniae and Mycoplasma pneumoniae [7, 8]. Droemann et al. found decreased TLR2 expression on alveolar macrophages from COPD patients and smokers [9]. It was also reported the expression of TLR2 on CD14 + monocytes in peripheral blood from stable COPD patients and healthy smokers were significantly decreased compare to healthy nonsmokers. In the same study the author showed a down-regulation of TLR2 was associated with reduced lung function parameters [10].

Inhaled corticosteroid is currently being suggested as the standard therapy in COPD guideline for severe or patients with recurrent exacerbations [11]. However, some epidemiological data showed an increased incidence of pneumonia which maybe related to long time use of combination therapy which included inhaled corticosteroids in COPD patients [12–14]. Gram positive bacteria like streptococcus pneumoniae, atypical pathogen like mycoplasma and chlamydia infections are major pathogens that cause community-acquired pneumonia and exacerbation of COPD [2, 4, 8, 15]. Therefore, long-term use of ICS in COPD patients could cause an inhibition of the innate immunity to infection should be examined. In one vitro study, Von Scheel et al. found that in bronchial epithelial cell culture, inhaled steroids budesonide (1.5-6 hours) did not influence TLR2 expression. But when co-stimulated with LPS, TNF, or dust together, budesonide increased TLR2 expression synergistically [16]. This result may reflected ICS role on the epithelial cell innate immune function during exacerbation status. But the long-term ICS use influence on airway local innate immunity had not been issued until now. In another study, the expression of TLR2 and CD14 on alveolar macrophages (AM) from COPD patients, smokers and non-smokers was reduced as compared to autologous monocytes. Comparing AM the author detected a reduced expression of TLR2 in COPD patients and smokers. In addition TLR2 mRNA and protein expression was increased after LPS stimulation on non-smokers AM in contrast to smokers and COPD patients [17]. In this retrospective study, we divided severe COPD patients into two groups according to whether they received inhaled corticosteroids for more than 1 year. Those patients in ICS naïve group were mostly treated in primary hospitals where ICS were not popular used in COPD patients. In order to minimize the bias, we selected subjects with the same age and same smoking history as well as the same gender in both groups. We observed that the TLR2 extracellular expression in the long-term ICS treatment group was significantly lower than the ICS naïve group. The TLR2 intracellular expression, TLR2mRNA and TNFαmRNA expression was also decreased in ICS long-term treatment group compare to ICS naïve group but this didn’t reached statistical significance. In Von’s study, it showed expression of TLR2 was lower on sputum neutrophils and soluble TLR2 (sTLR2) was higher in the supernatant in the COPD group. This may be one explanation of TLR2 expression difference between the surface and intracellular expression on the immune cells [6]. And we couldn’t exclude the contamination of neutrophil when use CD14 as the surface marker of macrophages. Our results suggest long-term use of ICS might have negative influence on the TLR2 expression in the airway macrophages of COPD patients. There was a strong relationship between the TLR2 extracellular and intracellular expression. But the mechanism caused the difference between TLR2 expression still needs to be elucidated.

The decreased TLR2 expression may be related to the inhibition of the transcription factor nuclear factor NF-κB activation pathway. The inhibition of NF-κB activation pathway is one of the main anti-inflammatory mechanisms of inhaled corticosteroid. At the same time, NF-κB activation is also the main pathway that innate immunity used against pathogenic microorganisms [18]. Both vitro and vivo studies showed that pathogen like streptococcus pneumonia and mycoplasma induce their pathogenic invasion through TLR2 activation, which is dependent on NF-κB pathway [14, 15, 18–21]. Therefore, the inhaled corticosteroid using the same pathway for anti-inflammatory effects may weaken the innate immunity to pathogen. In our study, a strong relationship between TNFα and TLR2 mRNA expression was observed. It was also reported that the expression of proinflammatory cytokine TNFα mRNA was strongly related to the NF-κB activation in asthma and COPD patients [18]. These results suggest the expression of both TNFα and TLR2 may be impaired by the same mechanism. Whether long-term use of inhaled corticosteroids which inhibits TLR2 expression, is related to NF-κB activation pathway still needs further investigation.

## Conclusion

We conducted this study in severe COPD male patients with heavy smoke histories in order to identify the effect of long term use of inhaled corticosteroid effect on TLR2 expression on macrophages from induced sputum. This study showed long-term use of ICS has a negative influence on TLR2 expression of COPD patients, which might be one explanation for increased pulmonary infection susceptibility.

### Summary at a glance

We compared TLR2 expression with flowcytometry and RT-PCR in severe male smoking COPD patients with or without long-time ICS treatment. The result showed TLR2 extracellular expression on the macrophages from induced sputum in long-term ICS treatment group was lower than the ICS naïve group.

## Authors’ original submitted files for images

Below are the links to the authors’ original submitted files for images. Authors’ original file for figure 1
Authors’ original file for figure 2
