# Supplementary figures and images for: Inhaled corticosteroid influence toll like receptor 2 expression in induced sputum from patients with COPD
Source: Transl Respir Med. 2013 Mar 19;1:7. doi: 10.1186/2213-0802-1-7 (PMC6733430; doi:10.1186/2213-0802-1-7)

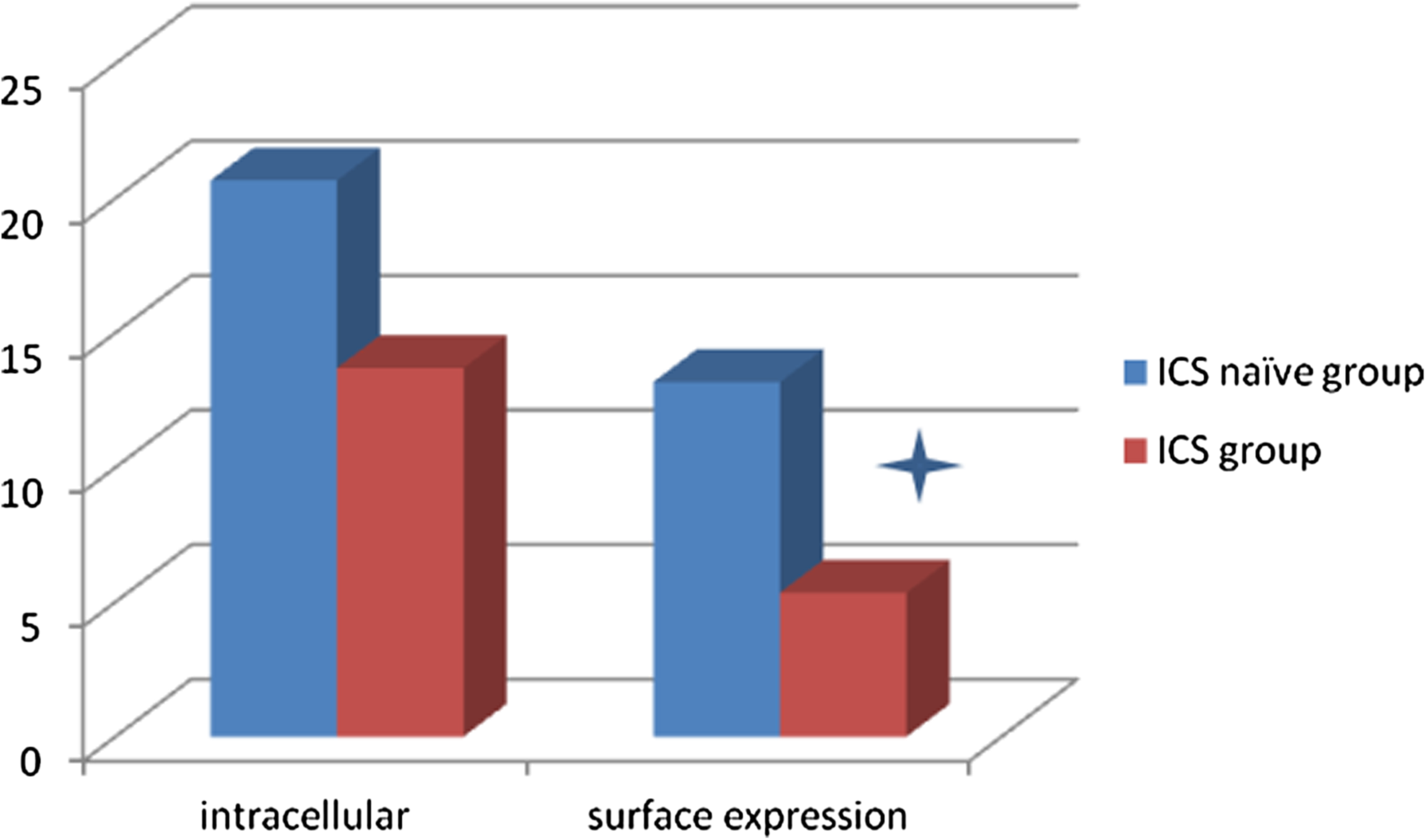

Supplement: Supplementary file 2 — Authors’ original file for figure 2 [file 40247_2012_6_MOESM2_ESM.tiff]
